# Sonographic Flow-Mediated Dilation Imaging versus Electronic EndoCheck Flow-Mediated Slowing by VICORDER in Pregnant Women—A Comparison of Two Methods to Evaluate Vascular Function in Pregnancy

**DOI:** 10.3390/jcm12051719

**Published:** 2023-02-21

**Authors:** Charlotte Lößner, Anna Multhaup, Thomas Lehmann, Ekkehard Schleußner, Tanja Groten

**Affiliations:** 1 Department of Obstetrics, University Hospital Jena, 07747 Jena, Germany; 2 Institute of Medical Statistics, Information Sciences and Documentation, University Hospital Jena, 07747 Jena, Germany

**Keywords:** pregnancy, hypertension, flow-mediated dilatation, flow-mediated slowing, vascular function

## Abstract

The evaluation of endothelial function is gaining interest and importance during pregnancy, since the impaired adaptation in early pregnancy has been associated with an increased risk in preeclampsia and fetal growth restriction. To standardize the risk assessment and to implement the evaluation of vascular function in routine pregnancy care, a suitable, accurate and easy to use method is needed. Flow-mediated dilatation (FMD) of the brachial artery assessed by ultrasound is considered to be the gold standard for measuring the vascular endothelial function. The challenges of the FMD measurement have so far prevented its introduction into clinical routine. The VICORDER^®^ device allows an automated determination of the flow-mediated slowing (FMS). The equivalence of FMD and FMS has not yet been proven in pregnant women. We collected data of 20 pregnant women randomly and consecutively while they presented for a vascular function assessment in our hospital. The gestational age at investigation was between 22 and 32 weeks of gestation, three had preexisting hypertensive pregnancy disease and three were twin pregnancies. The results for FMD or FMS below 11.3% were considered to be abnormal. Comparing FMD to FMS results in our cohort revealed a convergence in 9/9 cases, indicating normal endothelial function (specificity of 100%) and a sensitivity of 72.7%. In conclusion, we verify that the FMS measurement is a convenient, automated and operator-independent test method of endothelial function in pregnant women.

## 1. Introduction

Endothelial dysfunction is thought to be an important factor in the development of a placenta-associated disease such as preeclampsia, which is further associated with fetal growth restriction, chronic immune activation and multi-organ endothelial disease. In preeclampsia, the placenta secretes excess anti-angiogenic factors into the maternal circulation, leading to widespread endothelial damage and inflammation. Growing evidence links vascular dysfunction and the prediction of preeclampsia and fetal growth restriction in pregnancy. Diverse studies in high-risk women who developed preeclampsia have demonstrated that the endothelial dysfunction precedes the onset of clinical disease [1,2,3,4]. A recent systematic review of the vascular structure and function in preeclampsia demonstrates that an impaired endothelial function was consistently reported prior to, during and immediately after pregnancy, as evidenced by differences in the FMD of 1.7–12.2% [5]. Other publications verified that the FMD is decreased during a preeclamptic pregnancy [6,7]. Thus, measuring the cardiovascular function in obstetric routine care is increasingly discussed [8].

The endothelial function can be measured in coronary arteries and the periphery by measuring the vasomotor function after an intra-arterial infusion of pharmacologic substances, which enhance the release of endothelial nitric oxide. The disadvantage of these methods is their invasive nature, making them unsuitable for diverse studies. Celermajer et al. [9] developed the technique of flow-mediated dilatation (FMD) as a noninvasive method to measure the vascular endothelial function, and Coretti et al. [10] published the initial guidelines for the ultrasonic assessment of the FMD of the brachial artery. The method has been documented to correlate with the mentioned invasively assessed endothelial function in coronary arteries [11] and is considered today as the gold standard for measuring endothelial function [12]. However, there are various challenges in using the FMD measurement in daily clinical practice. The precision of measurement is highly dependent on the investigators’ experience and expertise, and the procedure is time and material-consuming. The VICORDER^®^ SMT medical device [13] mimics the FMD test procedure by analyzing flow-mediated slowing (FMS). The test allows for an automated and operator-independent testing of the endothelial function. The interchangeability of both test procedures has been shown [12,13,14], but an explicit investigation of pregnant women has not yet been done. 

In order to promote the introduction of the evaluation of the maternal hemodynamics into the routine care of pregnant women, we aimed to prove the equivalency of the FMD and FMS measurement in a cohort of pregnant women. We compared the FMD measuring by ultrasound to the FMS measuring by VICORDER^®^ in pregnant women for the first time.

## 2. Materials and Methods

During the assessment period between July 2020 and March 2021, pregnant women, scheduled for a routine assessment of cardiovascular function by FMD, received an additional measurement of FMS using VICORDER^®^. The women were consecutively included in our study. There were no exclusion criteria defined. In order to reduce the inter-observer variability the same examiner carried out all measurements. The clinical outcome data were collected from obstetric records following delivery. Ethical approval to include patient data for the analysis was obtained by the ethical committee of the Friedrich–Schiller University in Jena (2022-2683-Daten). The anonymous use of clinical data for research and educational purposes is covered by the governmental rules of Thuringia. 

For the FMD measurement, we used the ultrasound system Canon Aplio 500 (Canon Medical Systems GmbH, Neuss, Germany), which was equipped with a vascular software (Precision + APure +) for 2D-imaging (Preset Carotid), color and spectral Doppler, an internal electrocardiogram (ECG) monitor and a high-frequency vascular transducer. Our test subjects were in supine position with their arms in comfortable position for imaging the brachial artery. The artery was imaged above the antecubital fossa in a longitudinal plane. A segment with clear anterior and posterior intimal interfaces was chosen. To induce a flow stimulus, a conventional blood pressure cuff was placed on the patients’ forearm. The arterial occlusion was then created by a cuff inflation to the suprasystolic pressure (50 mmHg above systolic pressure) and held for 5 min. The occlusion caused ischemia and the consequent dilation of downstream resistance vessels via autoregulatory mechanisms. The cuff deflation then induced reactive hyperemia to accommodate the dilated resistance vessels. The resulting increase in shear stress caused the brachial artery to dilate. The brachial artery was measured at the same time in the cardiac circle by using ECG-gating during the image acquisition. We referred to the peak of the R-wave as the peak of systole, where the artery’s diameter is known to be at its largest state. Determined from the ultrasound image, the diameter of the brachial artery was compared before and after occlusion, and the percentage of change was determined. Measurements were performed in triplicates for each participant. We then referred to the respective mean value of the three measurements before and after vascular congestion.

To determine FMS, we used the VICORDER^®^ EndoCheck FMS Model. The test procedure was according to the device manual. The brachial pulse wave velocity (PWV) was measured simultaneously between the wrist and upper right arm over a measurement period of 10 min, where the occlusion time was 5 min. The slowing of PWV was determined continuously measuring minimal PWV in comparison to initial PWV value, the percentage of which is then called FMS.

**Figure 1:**
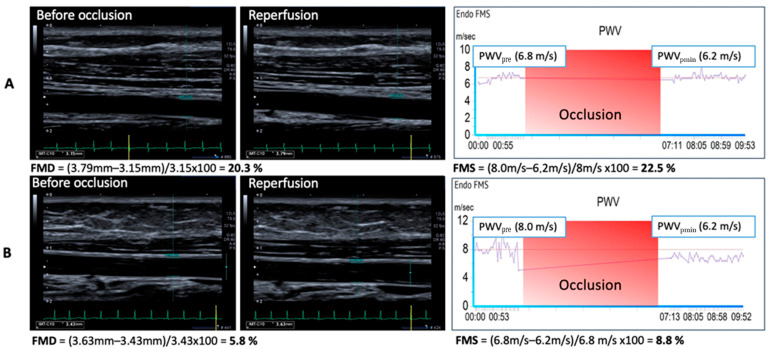
**Results of FMD and FMS determination for normal (A) and impaired (B) arterial stiffness.** For FMD measurement we used the ultrasound system Canon Aplio 500, which was equipped with a vascular software for 2D-imaging (Preset Carotid), colour and spectral Doppler, an internal electrocardiogram (ECG) monitor and a high-frequency vascular transducer. Diameter of brachial artery was ECG gated measured before and following arterial occlusion for 5 minutes. Flow mediated dilatation was calculated as percentage of change [(Diameter in mm following reperfusion − diameter in mm before occlusion)/diameter in mm before occlusion × 100]. To determine FMS, we used the VICORDER^®^ EndoCheck FMS Model. The brachial PWV was measured simultaneously between wrist and upper right arm. Occlusion was applied for 5 minutes (red phase) and PWV was determined continuously. FMS is calculated form the minimal value measured (PWVmin) in percent of the initial value [(PWV before occlusion − PWVpmin)/PWV before occlusion × 100].

As described by Shechter et al., we defined any FMD value > 11.3% as the normal endothelial function and, accordingly, any FMD value < 11.3% as the impaired endothelial function [15]. We defined the same clinical cut-off for the evaluation of FMS measurement results. Figure 1 shows results of FMD and FMS measurements indicating normal and impaired arterial stiffness (Figure 1).

Continuous baseline characteristics are summarized by the median and 25th/75th percentile, and absolute and relative frequencies are provided for categorical data. The diagnostic accuracy was assessed by sensitivity and specificity using the cut-off value of 11.3% for FMD and FMS measurements. Both measurements are described by the median and the 25th/75th percentile. The agreement of the two methods was assessed via Bland–Altman plot.

## 3. Results

### 3.1. Cohort Characteristics 

Descriptive data of our study population are presented in Table 1.

### 3.2. Comparison of Measured FMD and FMS Values 

Results for the FMD and FMS measurement and the calculated differences are listed for each of the 20 women in Table 2. The FMD values ranged from 5.5% to 15.7%, with a mean of 10.6%. The FMS values ranged from 8 to 17%, with a median of 15%. The difference between FMD and FMS value per test subject varied from 1.2% to 4.4%, with a median of 3.1%.

As shown in Table 3, all cases that identified to have a normal endothelial function by FMD measurement were also characterized to be normal by FMS, revealing a specificity of 100%. In 8 of 11 cases, where the FMD revealed a reduced endothelial function, both methods consistently showed values below 11.3% (sensitivity 72.70%). In 3 of 11 cases, the endothelial function was classified as reduced via FMD, but as normal by the FMS values. In all three cases, the FMD values were above 10% (cases 1, 2 and 19—see Table 2).

### 3.3. Agreement between FMD and FMS Method 

**Figure 2:**
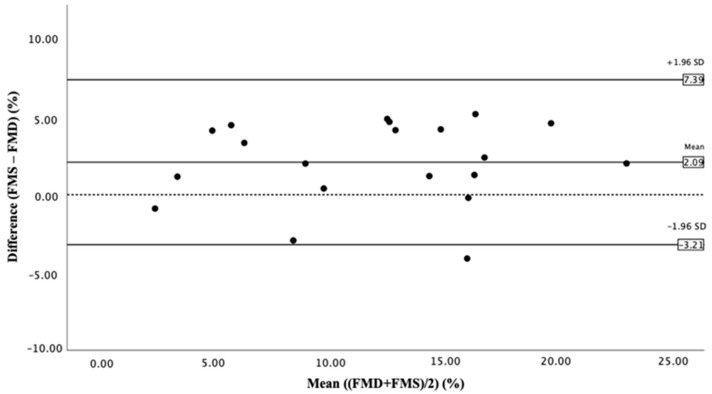
Bland–Altman plot of agreement between FMD and FMS method.

Figure 1 shows the Bland–Altman plot of agreement between the FMD and FMS method. As shown in the graph, the statistical limits were calculated by using the mean value and standard deviation of the difference between the two methods. The average discrepancy (the bias) between the FMD and FMS method lies at 2.09%. The 95% limits of agreement were 7.39% (bias + 1.96 SD) and −3.21 % (bias − 1.96 SD). A total of 19/20 data points are within ± 2 SD of the mean difference, as recommended by Bland and Altman. (Figure 2).

## 4. Discussion

Hypertensive pregnancy diseases are characterized by a general endothelial dysfunction, and the assessment of the endothelial function will play an increasingly important role in the medical care of these women. Increasing studies demonstrate that the determination of endothelial function by FMD is suitable for the prediction of pregnancy complications in high-risk pregnancies. In a systematic review of vascular structure and function in preeclampsia by Kirollos et al. [5] from 2019, an impaired endothelial function was consistently reported prior to, during and immediately after pregnancy, described by differences in the FMD of 1.7–12.2%. Diverse studies in high-risk women who developed preeclampsia have demonstrated that the endothelial dysfunction precedes the onset of clinical disease [3,4]. Other publications verified that FMD decreases during a preeclamptic pregnancy [6,7]. 

Additionally to the raising importance of endothelial function determination during pregnancy, the importance of evaluating the vascular function following pregnancies complicated by preeclampsia gains an increasing significance. Clinical observation demonstrates that women with a history of preeclampsia are known for higher cardiovascular morbidity and mortality later in life compared with controls, who had normotensive pregnancies [16]. The American Heart Association—Guidelines recognized preeclampsia as an important risk factor for cardiovascular diseases. This has led to the hypothesis that the endothelial status of these women is characterized by an early onset of aging. In 2018, Breetveld et al. [17] demonstrated that women with a history of preeclampsia remained associated with lower FMD years after pregnancy. 

However, in daily clinical practice, measuring FMD is challenging and did not prove to be suitable for routine care. Therefore, a method capable of being implemented in a routine setting and providing reliable and reproducible results on vascular endothelial function is urgently needed and is a fundamental requirement for interventional studies based on an altered endothelial function during pregnancy. The new technique to evaluate the endothelial function using the EndoCheck flow-mediated slowing (FMS) method allows for an automated and operator-independent determination of vascular function. It is based on the same pathophysiological principles as the FMD method. The comparability of both methods was shown before [12] but, so far, the data for pregnant women are missing. Our study provides this data.

We found a high concordance of FMD and FMS values in a cohort of 20 pregnant women. The Bland–Altmann analysis demonstrates that most data points were within ±2 SD of the mean difference, as recommended by Bland and Altman (Figure 2). The mentioned outliner (case 16—see in Table 1) showed the measurements of a healthy pregnant woman without pregnancy complications, where both the FMD and FMS count was above 11.3%.

As there is no generally valid cut-off specified in the literature up to date which distinguishes a normal from a reduced state of endothelial function, we defined the cut-off in our work according to the following study by Shechter et al. [15] from 2014. Herein, the usefulness of FMD to predict long-term cardiovascular events (all-cause mortality, nonfatal myocardial infarction, hospitalization for heart failure or angina pectoris, stroke, coronary artery bypass grafting and percutaneous coronary interventions) in subjects without heart disease was examined. A total of 618 subjects were divided into two groups: FMD ≤ 11.3% (*n* = 309) and FMD > 11.3% (*n* = 309), where 11.3% was the median FMD in that population. The groups were comparable regarding cardiovascular risk factors, lipoproteins, fasting glucose, C-reactive protein, concomitant medications and Framingham 10-year risk score. In a mean follow-up of 4.6 ± 1.8 years, 48 of 618 patients (7.7%) developed composite adverse cardiovascular events. All composite adverse cardiovascular endpoints were significantly more common in subjects with FMD below versus above the median FMD (15.2% vs. 1.2%, *p* = 0.0001). The statistical analysis demonstrated that the median FMD predicted cardiovascular events significantly and independently (*p* < 0.001) in healthy subjects with no apparent heart disease, in addition to those derived from a traditional risk factor assessment. In accordance, we defined a limit value for the clinical state of the endothelial function of 11.3%, considering that any FMD value > 11.3% indicates a normal endothelial function and, consequently, any FMD value ≤ 11.3% for a reduced endothelial function. In order to perform a comparison to the FMS method, we expected the same clinical cut-off for the FMS measurements. 

There was a convergence in both methods in 9/9 cases, indicating normal endothelial function (specificity of 100%). In 8/11 cases, a reduced FMD and FMS value was found for both methods (sensitivity of 72.70%). In 3/11 cases (27.30%), the endothelial function was measured as reduced via the FMD method, but as normal via the FMS-method. In those three cases, the FMD values were marginally lower than 11.3%, whereas the FMS values were above the cut-off (cases 1, 2 and 19—see Table 2). Case 1 shows a patient with FGR who suffered from pre-existent hypertension. She took anti-hypertensive medication, which might have affected the measurement. In case 2 and 19, pregnancies are described that were not complicated by the occurrence of any placental diseases. 

The major limitation of our study is the small number of pregnant women included. We aimed to include women consecutively in a nonselective approach. As a result of the non-selective approach, individual risk profiles showed large variations (BMI, maternal age, gestational age, parity, etc.). Fortunately, the high consistency of our results comparing the two methods was observed in all pregnant women regardless of their individual risk profile.

## 5. Conclusions

In conclusion, our data demonstrate the high accordance of FMD and FMS results. We could validate that the FMS measuring by the VICORDER^®^ is suitable for the routine care during pregnancy, yielding reliable clinical results. FMS is an electronic procedure viable for clinical screening and follow-up care in high-risk pregnancies, retrieving results equivalent to those obtained by FMD. We can confirm that large-scale prospective studies with a longitudinal observation of women throughout and after pregnancy to compare clinical outcome can be performed using the investigator independent automated method of the FMS measurement via VICORDER^®^ device. 

## Figures and Tables

**Table 1:** Outline of group characteristics and pregnancy outcome.

Group Characteristics	
BMI ^1^ (kg/m^2^)	25.9 (20.7/31.7)
Age (years)	33.5 (27.2/39.0)
Gestational age at measurement	26 + 5 (22 + 5/31 + 6)
Pre-existent hypertension	3 (15%)
Twin pregnancy	3 (15%)
History of placental disease	1 (5%)
**Clinical features at the time of measurement**	
None	2 (10%)
Cervical insufficiency	10 (50%)
FGR ^2^	4 (20%)
PROM ^3^	1 (5%)
Placenta previa	1 (5%)
Pathologic Doppler flow	1 (5%)
Orthostatic circulatory dysregulation	1 (5%)
Placental diseases at admission	4 (20%)
**Pregnancy Outcome**	
Gestational age at delivery	38 + 0 (34 + 4/39 + 0)
Mode of delivery	
Vaginal delivery	9 (45%)
Elective caesarean section	9 (45%)
Emergency caesarean section	2 (10%)
Placental diseases at delivery	6 (30%)
FGR ^2^ singleton pregnancy	2 (10%)
sFGR ^4^ twin pregnancy	2 (10%)
HELLP ^5^ and sFGR ^4^ twin pregnancy	1 (5%)
Eclampsia	1 (5%)
Premature birth	9 (45%)

Data are *n* (%) or median (interquartile range). ^1^ Body mass index; ^2^ fetal growth restriction; ^3^ premature rupture of membranes; ^4^ selective fetal growth restriction of one child in twin pregnancies; ^5^ hemolysis elevated liver enzymes and low platelet syndrome.

**Table 2:** Outline of measured FMD and FMS values.

Comparison of Values per Test Subject			
Patient No	FMD (%)	FMS (%)	Difference % (FMS–FMD)
1	10.13	15.00	+4.87
2	10.32	15.00	+4.68
3	3.53	8.00	+4.47
4	15.61	18.00	+2.39
5	4.67	8.00	+3.33
6	21.99	24.00	+2.01
7	8.00	10.00	+2.00
8	12.80	17.00	+4.20
9	17.41	22.00	+4.59
10	2.89	2.00	−0.89
11	2.88	7.00	+4.12
12	9.94	7.00	−2.94
13	15.73	17.00	+1.27
14	2.84	4.00	+1.16
15	16.20	16.00	−0.20
16	18.10	14.00	−4.10
17	13.80	15.00	+1.20
18	9.60	10.00	+0.40
19	10.85	15.00	+4.15
20	13.82	19.00	+5.18

Data for FMD were mean of triplicates performed.

**Table 3:** Comparison of classification of endothelial function by FMD or FMS using the cut off < 11.3% to determine impaired function.

			FMD		Total
			>11.3% Indicating Normal Endothelial Function	<11.3% Indicating Reduced Endothelial Function	
FMS	>11.3% indicating normal endothelial function	Count	9	3	12
		% of reduced FMD	100.0%	27.3%	60.0%
	<11.3% indicating reduced endothelial function	Count	0	8	8
		% of reduced FMD	0.0%	72.7%	40.0%
Total		Count	9	11	20
		% of reduced FMD	100.0%	100.0%	100.0%

## Data Availability

All data retrieved are displayed in this manuscript.
